# Supplementary figures and images for: Establishment of a congenic strain for the oyster mushroom reveals the structure and evolution of mating-type loci
Source: PLoS Genet. 2026 Apr 8;22(4):e1011966. doi: 10.1371/journal.pgen.1011966 (PMC13108863; doi:10.1371/journal.pgen.1011966)

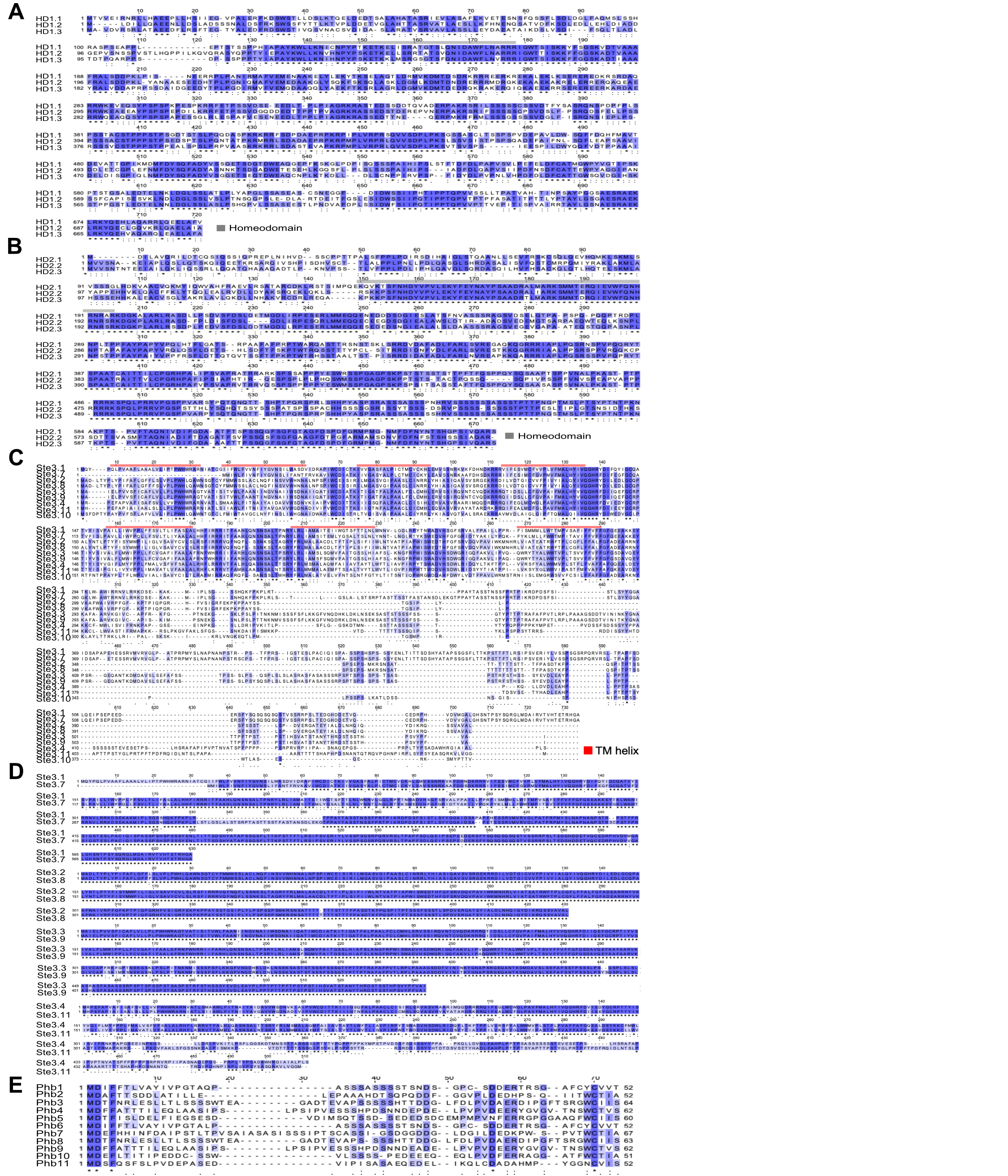

Supplement: S1 Fig — Alignment of (A) HD1, (B) HD2, (C and D) Ste3, and (E) Phb proteins from strains PC9 and PC15. Three levels of shading indicate degrees of sequence similarity: dark blue background with an asterisk (*) indicates identical amino acids, intermediate blue background with a colon (:) indicates conserved amino acids, and light blue with a dot (.) indicates semi-conserved amino acids. Grey and red shading indicate the homeodomain regions in HD proteins and the transmembrane (TM) helix regions in Ste3 proteins, respectively. (TIFF) [file pgen.1011966.s001.tiff]

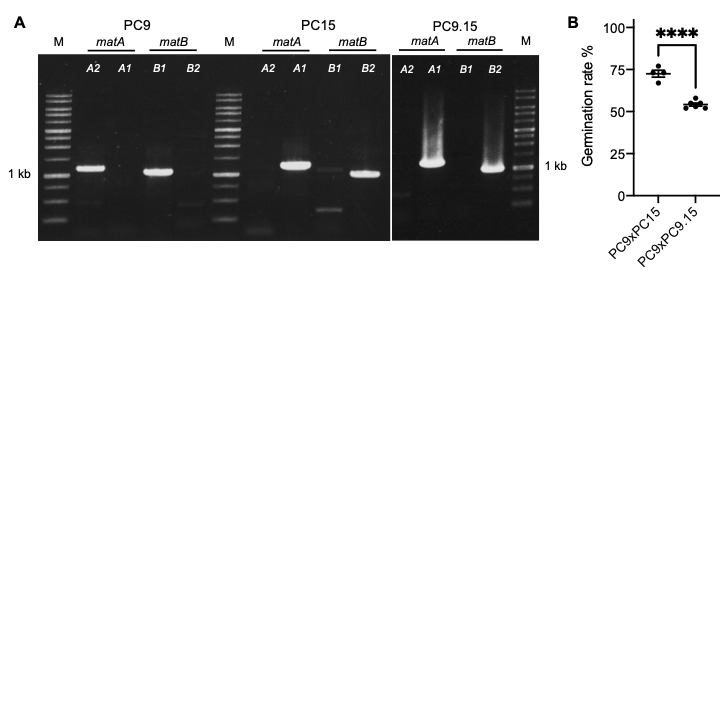

Supplement: S2 Fig — (A) PCR amplification of the matA and matB loci in PC9, PC15, and the congenic strain PC9.15 using mating-type specific primers: PC9-matA-F/R, PC9-matB-F/R, PC15-matA-F/R, and PC15-matB-F/R. (B) Germination rates of PC9 x PC15 and PC9 x PC9.15. Statistical significance was calculated using a two-tailed unpaired Student’s t-test. (TIFF) [file pgen.1011966.s002.tiff]

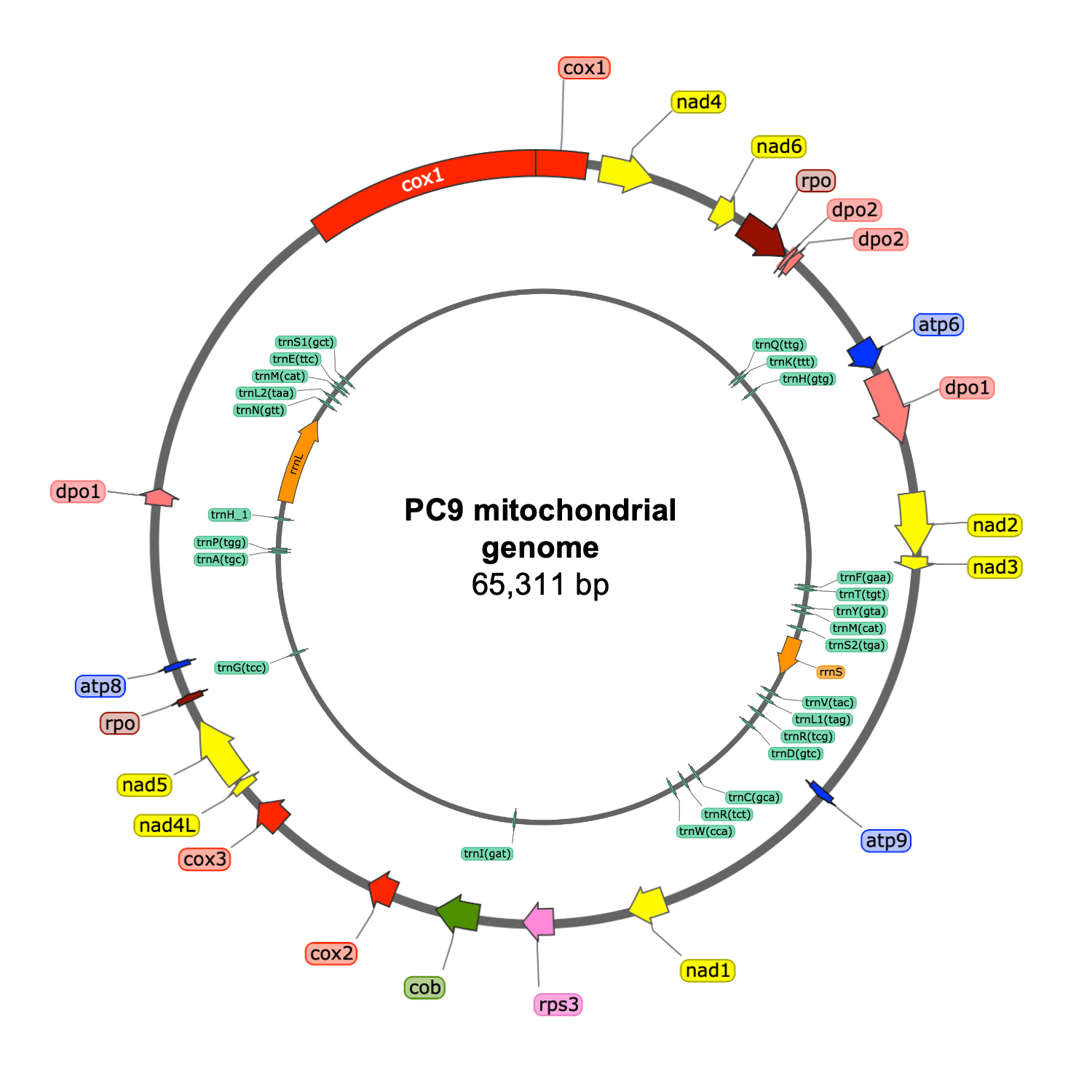

Supplement: S3 Fig — Tracks (outer to inner) show: (1) annotation of mitochondrial DNA-encoded genes: subunits of cytochrome c oxidase/complex IV (red), NADH dehydrogenase/complex I (yellow), ATP synthase/complex V (blue), apocytochrome b cob (green), ribosomal small subunit protein rps3 (pink), DNA polymerase dpo (salmon) and DNA-directed RNA polymerase rpo (brown); (2) annotation of RNA genes: ribosomal RNA genes rrnS and rrnL (orange) and 25 transfer RNA (green). (TIFF) [file pgen.1011966.s003.tiff]

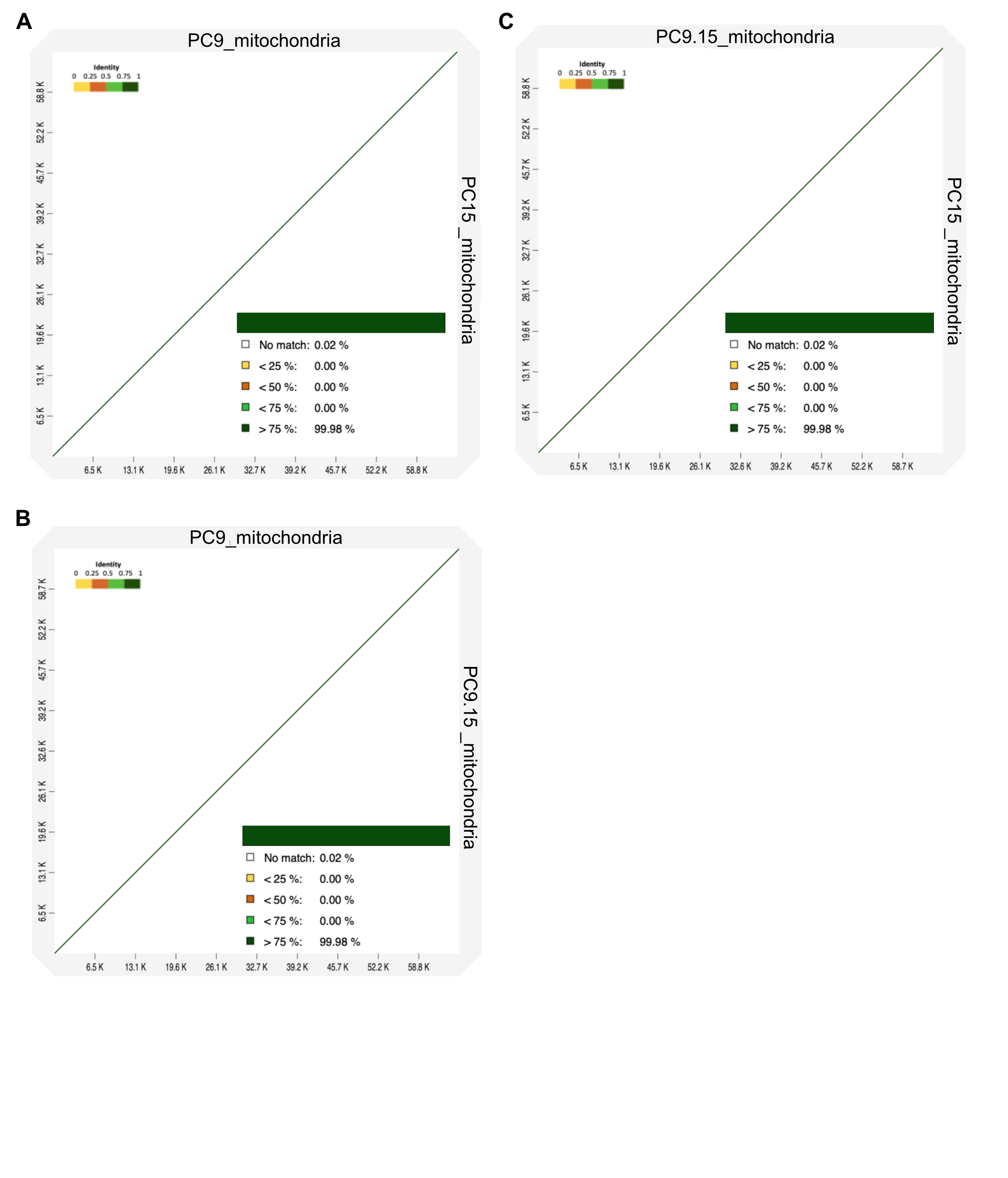

Supplement: S4 Fig — Dot-plot alignment generated by D-Genies minimap v2.24 showing the mitochondrial genomes of P. ostreatus strains: (A) PC9 (target) vs. PC15 (query), (B) PC9 (target) vs. PC9.15 (query), and (C) PC9.15 (target) vs. PC15 (query). Percentages indicate the fraction of the genome participating in alignments with >75% sequence identity. (TIFF) [file pgen.1011966.s004.tiff]

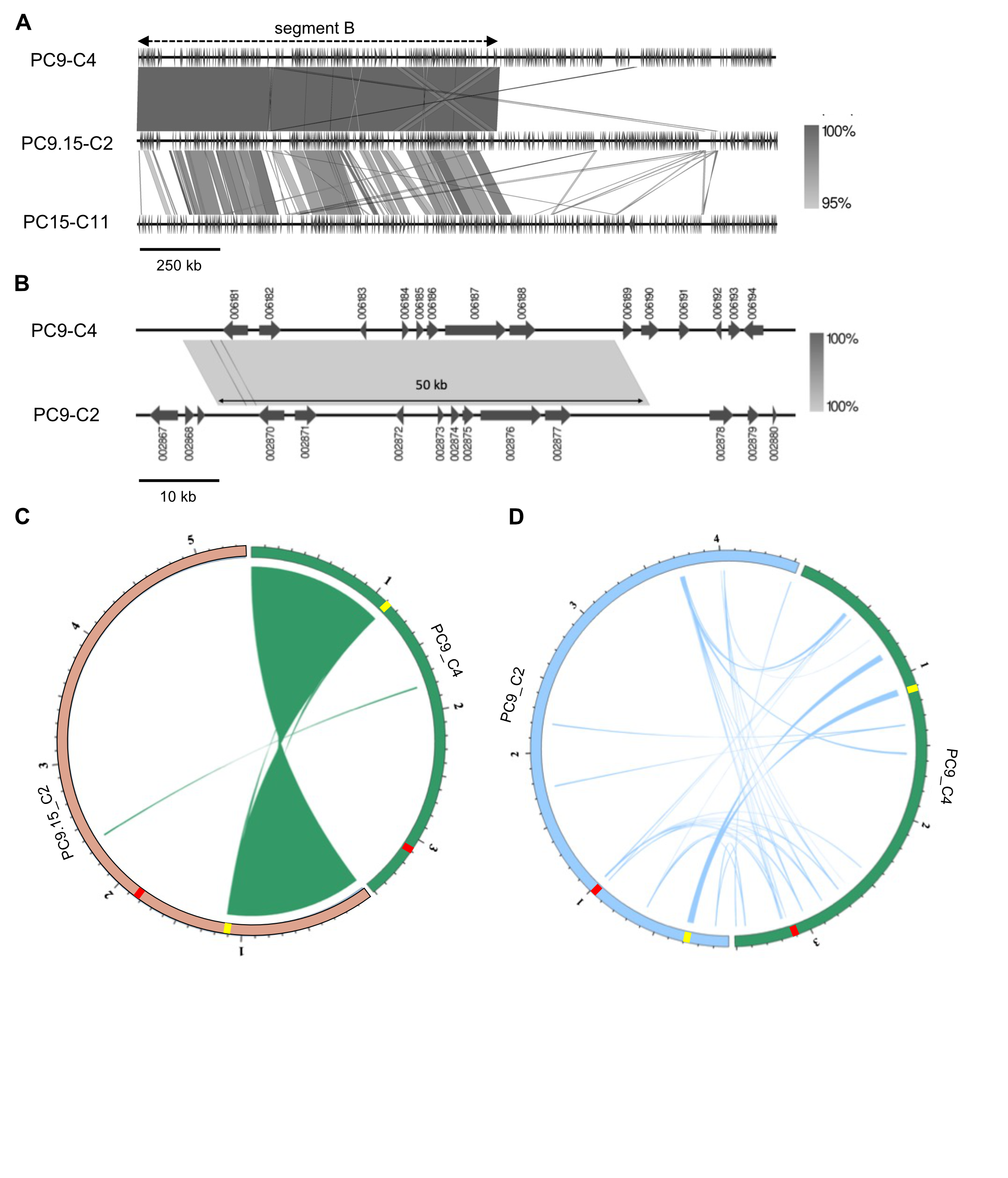

Supplement: S5 Fig — (A) Partial sequence alignment of PC9 chromosome 4 (PC9_C4), PC9.15 chromosome 2 (PC9.15_C2), and PC15 chromosome 11 (PC15_C11). (B) Partial sequence alignment of PC9 chromosome 4 (PC9_C4) and PC9 chromosome 2 (PC9_C2). (C and D) Circos plots showing regions of high similarity (identity > 95%, length > 10kb) between PC9 chromosome 4 (PC9_C4) and PC9.15 chromosome 2 (PC9.15_C2) (C) and PC9 chromosome 4 (PC9_C4) and PC9 chromosome 2 (PC9_C2) (D). Red boxes represent centromere region and yellow boxes represent segment B. The letter “C” indicates the chromosome. (TIFF) [file pgen.1011966.s005.tiff]
