# Supplementary material for: Establishment of a congenic strain for the oyster mushroom reveals the structure and evolution of mating-type loci
Source: PLoS Genet. 2026 Apr 8;22(4):e1011966. doi: 10.1371/journal.pgen.1011966 (PMC13108863; doi:10.1371/journal.pgen.1011966)
Supplement: Attachment — Submitted filename: Response to Reviewers.pdf [file pgen.1011966.s014.pdf]

Dear Geradine,

Thank you so much for handling our manuscript! We would like to thank the reviewer for the helpful and constructive comments. We have revised the manuscript accordingly, and all changes are tracked in the revised version. Below we provide a detailed, point-by-point response to the reviewer's comments.

Sincerely,

Ping

Reviewers' comments:

Reviewer #1: This is an interesting and well-structured manuscript that reports substantial progress towards facilitating molecular genetic analysis in the oyster mushroom Pleurotus ostreatus. This species is of interest as an experimental system, both because of its importance in agriculture as a food organism, and from a basic science perspective because of its ability to prey on nematodes.

The background to the work is that P. ostreatus was known to have a tetrapolar mating system involving two unlinked genetic loci (matA and matB) that have multiple (>10) alleles each. A pair of P. ostreatus lab strains, called PC9 and PC15, that can mate with each other was previously developed. However, PC9 and PC15 have quite different phenotypes (PC9 grows much faster) and there is substantial DNA sequence divergence between their genomes, which makes them unsuitable as a pair of parents for use in genetic mapping experiments. In the current manuscript, Lee et al have done two main things. (1) They identified and annotated the genes in the matA and matB loci, and examined the high sequence and structural diversity of these loci among ~10 isolates of P. ostreatus. (2) By repeated backcrossing, they introduced the matA and matB alleles from PC15 into the PC9 genetic background, to create a new strain called PC9.15; they show that PC9.15 and PC9 have similar phenotypes and can mate together. By genome sequencing, they show that the PC9.15 genome is almost identical to the PC9 genome except for islands of 300-1000 kb around the two introgressed mat loci, and some minor unplanned rearrangements. Therefore, PC9 and PC9.15 are suitable for use as parental backgrounds for future genetic analyses in P. ostreatus, such as mapping genes required for nematode killing. The manuscript reports an extensive body of work that has been done to a high standard. It makes a significant step forward for oyster mushroom, and for our understanding of mating-type locus population genetics and evolution in basidiomycete fungi.

I note that the manuscript mentions that the structure of the matA locus of P. ostreatus was previously reported by another group (Shnyreva and Shnyreva, 2023; ref. 13). I read this paper, and my opinion is that the new manuscript is far superior. The Shnyreva and Shnyreva paper did not examine the matB locus at all; their annotation overlooked one of the HD genes in the matA locus; they did not examine mat locus sequence/structure diversity with the species P. ostreatus; and they did not construct a pair of congenic mating strains. I think that Lee et al have given appropriate credit to it.

Major comment

L200-216: It's not clear what criteria the authors use to differentiate between "distinct alleles" and "the same allele". For example L202 says that "SS14 shares an identical matA allele with PC9 (allele A2)". Does this mean identical nucleotide sequences of the whole matA locus, or just identical amino acid sequences of HD1.1 and HD2.1? Does each of the different HD1.x and HD2.x names in Figure 2 correspond to a different amino acid sequence?

R: We thank the reviewer for these questions. SS14 carries a *matA* allele identical to that of PC9, with complete nucleotide and protein sequence identity across the entire *matA* locus (L228-230). Yes. Each HD1.x and HD2.x designation in Figure 2 corresponds to a distinct amino acid sequence. We have added clarifying sentences to explicitly define how alleles and *HD* gene variants are distinguished (L219-221).

Minor comments

There seems to be an error or contradiction regarding levels of sequence divergence in Ste3 proteins, between the data in Table S2 and the corresponding text on L175-178. Instead of using the terms "highly similar" and "considerably more divergent", it would be more informative to state the actual percentage identities.

R: We thank the reviewer for identifying the errors in Table S2. We have corrected these errors and also stated the specific amino acid identity percentage in the main text now (L194-197).

There also seem to be inconsistencies in the presence/absence of links connecting genes in Figure 1A. The legend says that links are drawn if sequence identity is >90%. However, Ste3.3 vs Ste3.9 is 96.9% identical (Table S2) but they are not linked. I wonder if some of the gray genes should also have links, e.g. the two gray genes between PHB1 and STE3.1 in PC9 seem to have two orthologs in PC15 (Figure 1A).

R: We thank the reviewer for these questions. Yes—Ste3.2 vs Ste3.8, Ste3.3 vs Ste3.9, and several of the genes shown in grey should also be linked. To improved clarity, we increased the identity threshold for drawing links to > 95% and updated Figure 1 accordingly. Orthologous genes, including those shown in grey, are now consistently linked if their sequence identity is over 95% (Fig 1, 2, and 6).

Assembly version and accession numbers need to be stated, both for the pre-existing genome sequences and for the new data, in Tables 1 and 2 and in some of the supplementary tables. For example, Table S3 contains lists of contig names and gene coordinates, but without accession numbers. On Line 137, please cite a publication and an assembly accession number for "the JGI genome".

R: We thank the reviewer for this comment. We have added genome assembly accession information for all strains in Table 1. For Table S3, we clarified the assembly accessions in the table legend, noting that the gene annotations were generated in this study. We also

added the appropriate publication reference and assembly accession number for the PC15 JGI genome (L144).

Figure 2A has two separate parts that are referred to at different places in the text, and so does Figure 2B. It would be better to call the 4 parts A-D.

R: In line with reviewer's suggestion, we reorganized the original Figure 2 to improve readability and make it easier to follow the corresponding text and results. For consistency, we have also revised Figure 1 to comprise four panels labeled A-D.

L32, say that there were 10 rounds of backcrossing.

R: We have added the "Using of 10 rounds of genetic backcross" in Line 32.

L145: Cite Figure 1A here.

R: As Figure 1 has been reorganized into panels A-D, we have updated the citation accordingly and now refer to Figure 1B in Line 152.

L147: Do you mean "the matB locus in many basidiomycetes is bordered…" ?

R: We thank the reviewer for pointing out the ambiguity in our original wording. We have revised the sentences to make it clear (L154-158).

L304: Delete the phrase "and enhanced performance of newer funnotate versions", because there is a still 2000-gene difference between the PC9.15 annotation and the PC9 annotation when the same software version is used.

R: We have deleted the phrase as suggested.

L306-309: I don't think that the term "sequence similarity" is being used correctly in these sentences. I think that perhaps the numbers 99.47% and 56.31% refer to the percentage of the length of one genome that can be aligned to another genome, in alignments that have >75% sequence identity.

R: Yes. The reviewer is correct that the reported values refer to the proportion of the reference genome covered by whole-genome alignments at >75% nucleotide identity, rather than an overall per-base sequence similarity metric. We have revised the text accordingly, replacing "sequence similarity" with wording that explicitly describes genome-wide alignment coverage to clarify the results (L346-350).

L346-359 and Figure 6: The section about the mitochondrial genome seems unnecessary and unrelated to the rest of the manuscript. I suggest deleting it except for the final sentence (L358), which can be moved to L320.

R: We agree with the reviewer and have removed this section, retaining only the final sentence, which has been relocated to Line 375-379 and move the related figures and tables to the Supplementary materials.

L372: I'm not sure why the authors suggest that mating regulation system in P. ostreatus is "more flexible"? Do you mean more flexible than in Coprinus cinereus? The P. ostreatus and C. cinereus systems seem to be very similar (they both have more PHB genes than STE3 genes), so I don't understand why P. ostreatus is considered more flexible.

R: We thank the reviewer for raising these questions. We agree that the term "more flexible" was unclear and could be misleading, so we have therefore removed this wording and revised the sentence (L423-425).

L415: Can we be certain that recombination is suppressed in these regions around the mat loci? I agree that the regions seem large, but are they larger than what is expected by chance, given the number of backcrosses and the recombination rate in P. ostreatus?

R: We thank the reviewer for these questions. We agree that the size of the flanking regions alone is not sufficient to conclude recombination suppression. Using the *P. ostreatus* linkage map (~35 kb/cM), the ~1.13 Mb (*matA*) and ~300 kb (*matB*) intervals correspond to ~32.1 cM and ~8.5 cM, respectively. Over 10 backcross generations, the probability of at least one recombinant occurring within these intervals is ~96% for *matA* and ~59% for *matB*, suggesting that the persistence of the larger *matA* flanking region is consistent with the possibility of reduced recombination. We have revised the discussion accordingly (L493-505).

Figure 2B2: It would be helpful to highlight the Ste3 genes from PC9 and PC15 in red and yellow, as in Figure 1B.

R: We thank the reviewer for this suggestion. We add the color in Figure 2C and 2D to highlight the HD and Ste3 genes from PC9 and PC15.

L382 typo: cinereus

L608 typo: "(A)" is missing.

R: Thank you. We have corrected the typos.
